# Social Support is Associated with Better Health in the Face of COVID-19

**DOI:** 10.1093/geroni/igab046.2747

**Published:** 2021-12-17

**Authors:** Laura Bernstein, Julie Hicks Patrick

**Affiliations:** West Virginia University, Morgantown, West Virginia, United States

## Abstract

The COVID-19 pandemic has the potential to influence the health of the nation, both directly and indirectly, though increased stress. As with other stressful crises, social support may buffer against the deleterious effects of the stress surrounding COVID-19 (Cohen & Wills, 1985). We were interested in how self-reported health changed during the first year of the COVID-19 pandemic in the United States and whether age or positive social exchanges influenced this potential change. We tested a latent growth curve model of change in SF12 scores over 4 points of measurement during the first year of the pandemic. Data from 237 adults (Mean age 40.7 yrs) were used to test whether SF12 scores changed over the 11 month period and whether age and initial positive social exchanges influenced both the intercept and trajectory of change over time. Results showed that the model fit the data well, X2 (DF = 13, N = 237) = 11.44, p = .57, RMSEA < .06. Of note, older age was associated with both better initial health (b = .036**) and a slower decrease over the year (b = -.005*). Initial positive social exchanges were associated with better initial health (b = .067*) but did not alter the trajectory of change over time. These findings suggest an age-related advantage for health in the face of COVID-19 and that positive social support is associated with better health, at least at the very beginning of the pandemic.

